# Heterologous prime-boost immunization of two-component vaccine candidate PWDVax protected pigs against F18 enterotoxigenic *Escherichia coli* post-weaning diarrhea

**DOI:** 10.1128/iai.00406-24

**Published:** 2025-03-12

**Authors:** Chongyang Zhang, Siqi Li, Ipshita Upadhyay, Sai Simah Reddy Vakamalla, Kathyrn L. Lauder, Chance Hansen, Kristen Ann Massey, Courtney Hayes, Nicole L. Herndon, Weiping Zhang

**Affiliations:** 1 Department of Pathobiology, University of Illinois at Urbana-Champaign 14589 , Urbana, Illinois, USA; 2 Department of Clinical Veterinary Medicine, University of Illinois at Urbana-Champaign 14589 , Urbana, Illinois, USA; 3 Animal Care Program for Laboratory Animals, Division of Animal Resource, University of Illinois at Urbana-Champaign 14589 , Urbana, Illinois, USA; Universite de Geneve, Genève, Switzerland

**Keywords:** post-weaning diarrhea (PWD), vaccine, PWDVax, enterotoxigenic *Escherichia coli *(ETEC), F18 ETEC, heterologous prime-boost immunization

## Abstract

Post-weaning diarrhea (PWD) is associated predominantly with enterotoxigenic *Escherichia coli* (ETEC) and continuously causes significant economic losses to swine producers worldwide. Currently, there are no effective countermeasures against this significant swine disease. Challenges persist in developing vaccines against PWD since ETEC strains produce heterogeneous virulence factors, including F4 (K88) and F18 fimbria and heat-labile toxin (LT), heat-stable toxin type I (STa), heat-stable toxin II (STb), and Shiga toxin type 2e (Stx2e, also causes edema disease). An effective PWD vaccine would induce broadly protective immunity, ideally against two fimbriae and four toxins. In this study, by applying a novel epitope- and structure-based multiepitope-fusion-antigen (MEFA) vaccinology platform, we created a monomeric polyvalent fimbria-toxin protein (fimbria-toxin MEFA) and a holotoxin-structured protein to target PWD virulence determinants (F4 and F18 fimbriae and LT, STa, STb, and Stx2e toxins) and developed a two-component multivalent PWD vaccine candidate, PWDVax. We further applied a heterologous prime-boost immunization strategy and assessed vaccine protection against F18 ETEC-associated PWD. Piglets, after being primed intramuscularly with a fimbria-toxin MEFA monomer protein and boosted orally with live *Escherichia coli* bacteria producing GM_1_-binding holotoxin-structured fimbria-toxin MEFA, developed IgG and secretory IgA responses to the target fimbriae and toxins. Challenged with an F18ac ETEC strain, the immunized piglets were protected against watery diarrhea (87.5%) or any diarrhea (66.7%). These data indicated that PWDVax protects against F18 ETEC-associated PWD and can become an effective PWD vaccine. The two-component vaccine and heterologous prime-boost immunization strategy may be instructive for developing neonatal vaccines in general.

**IMPORTANCE:**

Enterotoxigenic *Escherichia coli* (ETEC)-associated post-weaning diarrhea (PWD) is a global swine disease, remains a major threat to pig health and well-being, and causes significant economic losses. Currently, there are no effective vaccines available against this disease because of challenges including heterogeneity among ETEC strains (or virulence factors) and difficulties in inducing protective immunity against some key virulence determinants. PWDVax, a two-component PWD vaccine candidate, unprecedentedly targeted two ETEC fimbriae (F4 and F18) and four toxins (LT, STa, STb, and Stx2e), the virulence factors associated with nearly all PWD clinical cases. Under a heterologous prime-boost immunization schedule, it induced broad systemic and mucosal antigen-specific antibodies but also protected weaned piglets against F18 ETEC diarrhea. This makes PWDVax potentially an effective vaccine to protect against PWD, particularly the current F18 ETEC-associated severe PWD outbreaks in the United States. Additionally, the two-component vaccine and heterologous prime-boost immunization strategy may also facilitate the development of effective neonatal vaccines for humans.

## INTRODUCTION

Post-weaning diarrhea (PWD) is a common disease for newly weaned piglets (1). Unlike suckling piglets that can be protected by passive maternal antibodies from the mothers immunized with enterotoxigenic *Escherichia coli* (ETEC) fimbriae and toxoid antigens or naturally infected with ETEC bacteria, weaned piglets become vulnerable to ETEC infection and can develop watery diarrhea at the moment of entering weaning. PWD results in slow growth, weight loss, and acute death and requires prophylaxis and/or metaphylaxis treatments and extra management attention, leading to significant economic losses to swine producers worldwide (2, 3). Early studies estimated that PWD can lead to the death of up to over 2% of weaned pigs (4–6), particularly in swine farms with a history of high mortality (7). A case study in the year 2000 estimated that the overall economic losses from PWD were $3.13–$5.88 per nursery pig (3). In the United States, there are around 70 million pigs produced annually in recent years (8); even based on a minimum $3 loss per pig (by ignoring the inflation and significant price increase of nursery pigs over the last 20 years), the annual loss for the US swine producers can be over $200 million, and more than $2 billion worldwide based on the annual global production of over 700 million pigs (9).

PWD can be caused indirectly by factors including separation from the sows, particularly depletion of maternal antibodies, stress from co-mingling with piglets from other litters, and diet change. The direct cause, however, is the infection of viral pathogens, including rotaviruses, transmissible gastroenteritis viruses and porcine epidemic diarrhea viruses, parasitic pathogens, and mostly diarrheal bacteria, particularly *Escherichia coli*. Among the diarrheal *E. coli* bacteria, enterotoxigenic *E. coli* (ETEC), a group of *E. coli* that produce enterotoxins, is the predominant cause of PWD (4, 10).

ETEC strains causing diarrhea in pigs produce two major types of virulence determinants: fimbria and toxin. Fimbria facilitates ETEC bacterial attachment to host receptors on pig intestinal epithelial cells and colonization in pig small intestines (11). Fimbriae F4 (K88), F5 (K99), F6 (987P), F7 (F41), and F18 are often detected from ETEC strains isolated from pigs with PWD (12–16). Among these fimbriae, F4 and F18 are the most prevalent, and ETEC strains producing F4 (F4ac) or F18 (F18ac) fimbria are responsible for a vast majority of PWD cases (15), including over 95% of the PWD clinical cases in the United States (16). Toxins produced by the colonized ETEC bacteria react with intestinal epithelial cells to disrupt homeostasis, causing hypersecretion of water and electrolyte fluid into the gut lumen and resulting in diarrhea (17). Toxins produced by ETEC strains isolated from pigs with PWD are heat-labile toxin (LT), heat-stable toxin type I (STa), heat-stable toxin type II (STb), enteroaggregative heat-stable toxin type 1 (EAST1), and Shiga toxin 2e (Stx2e) (15, 16, 18, 19). Toxin EAST1 is not necessary for causing diarrhea in pigs (20, 21), and Stx2e is more associated with edema disease (ED) (22, 23), but toxins LT, STa, and STb are the virulence determinants in causing diarrhea in pigs (24, 25). An early study revealed that F4 or F18 fimbrial ETEC isolates producing toxins LT/STb, LT/STa/STb, or STa/STb/Stx2e are the major pathovars for PWD in the United States (16). The recently emerging F18ac ETEC strains that cause more severe PWD and are associated with higher morbidity and mortality rates in the US swine production systems, however, produce four toxins, LT, STa, STb, and Stx2e, and are largely resistant to antibiotics (26).

A vaccine that induces broad protective immunity against ETEC fimbriae and enterotoxins has been considered preferable against ETEC diarrhea (10, 27, 28). Unfortunately, there are no effective vaccines currently available against ETEC-associated PWD. Products containing live *E. coli* bacteria that express F4 and/or F18 fimbria are used as PWD vaccines in some regions or countries; studies suggested that one product can reduce PWD severity or shorten diarrheal duration against infection from an F4 or F18 ETEC strain (29, 30). However, unlike an avirulent F4-fimbrial *E. coli* field isolate or recombinant strain that can induce F4-specific functional antibodies and protect pigs against F4 ETEC bacteria intestinal colonization or diarrhea (29, 31–33), live F18-fimbrial bacteria or purified F18 fimbriae were shown ineffective in inducing F18-specific functional antibodies and did not protect against F18 ETEC infection (34, 35), suggesting vaccine products with bacteria producing F18 fimbriae are not effective in eliciting protective immunity against PWD, particularly the F18 ETEC-associated PWD which is the major threat currently to the US swine production. The F18 fimbrial adhesive minor subunit, FedF, on the other hand, can induce protective immunity against F18 adherence or F18-fimbrial ETEC bacteria intestinal colonization (33, 36). Additionally, none of the current cellular vaccine products carry toxin antigen components to induce neutralizing antibodies against ETEC toxins, which essentially stimulate fluid hypersecretion and cause watery diarrhea in pigs or other hosts.

Another major challenge is to develop a PWD vaccine to elicit active immunity in piglets by the time of entering weaning, at the age of 3 or less than 3 weeks, to protect against ETEC infection. This requires a PWD vaccine to be administered to piglets at a very young age and to induce protective anti-fimbria and antitoxin immunity in piglets before they are weaned. Therefore, an effective PWD vaccine needs to overcome the challenges of virulence heterogeneity among ETEC strains and the early development of active and protective immunity in young piglets. Realizing that we cannot simply mix the fimbria and toxin antigens for a safe and protective PWD vaccine product, we recently applied a novel epitope- and structure-based multiepitope-fusion-antigen (MEFA) vaccinology platform and constructed epitope-based polyvalent fimbria-toxin protein immunogens to induce functional antibodies against F4 and F18 fimbriae but also LT, STa, STb, and Stx2e toxins (37, 38). In this study, we created a new monomeric fimbria-toxin MEFA protein and a GM_1_-binding AB_5_ holotoxin-structured fimbria-toxin MEFA, used the monomer protein as an acellular component and the AB_5_ holotoxin-structured fimbria-toxin MEFA to be expressed by live *E. coli* bacteria as a cellular component, and constructed a novel two-component PWD vaccine candidate. We further applied a heterologous prime-boost vaccination strategy to induce protective immunity in young piglets and evaluated the efficacy of the vaccine candidate against PWD in a challenge study with an F18ac ETEC strain that was isolated from the current F18 ETEC PWD outbreak in the United States (26). Data showed that the immunized piglets developed antigen-specific antibody responses and were protected against the F18 ETEC diarrhea, indicating the potential application of this candidate as an effective PWD vaccine.

## RESULTS

### PWDVax, a two-component multivalent vaccine candidate for PWD

**Fig 1:**
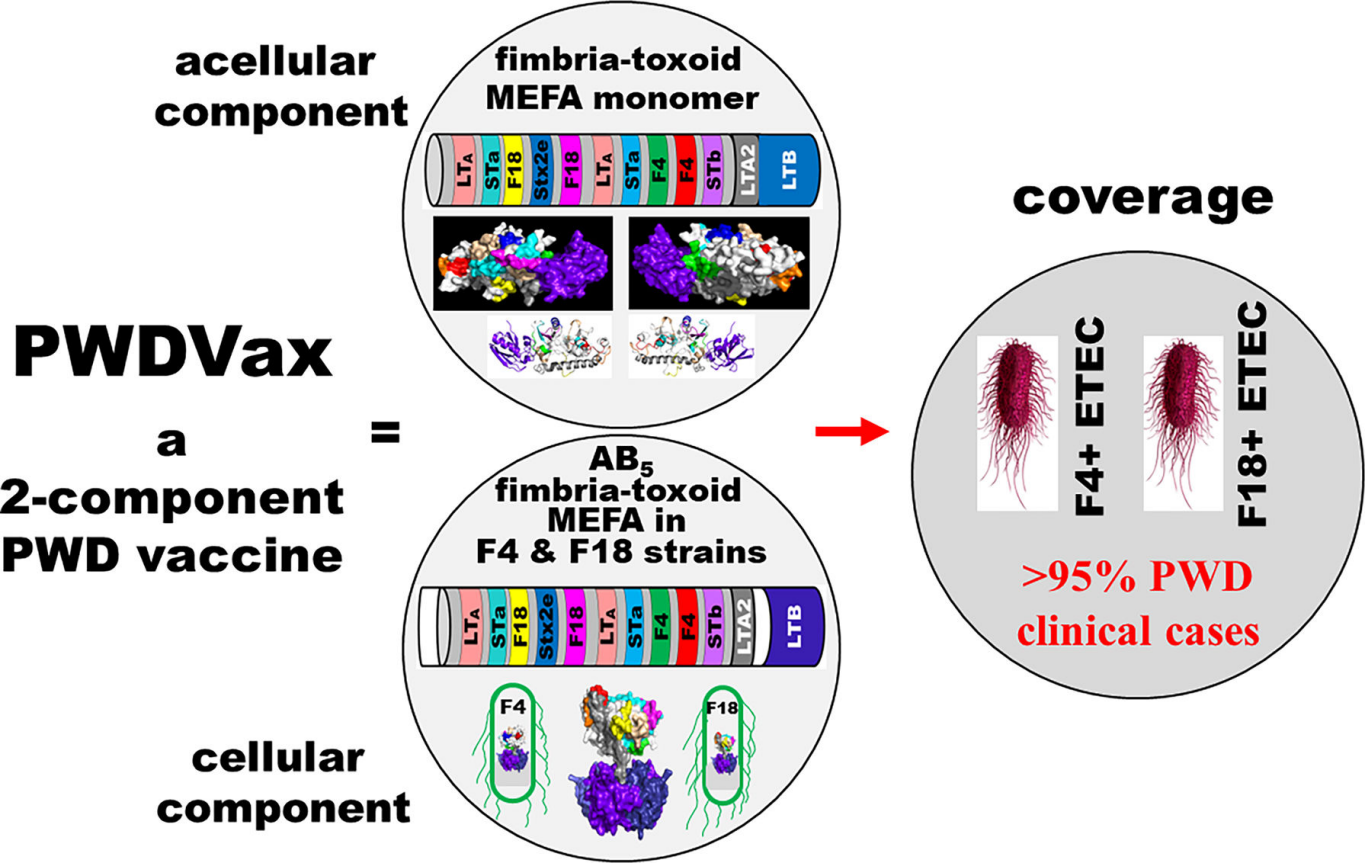
Diagram of PWD vaccine candidate PWDVax and the vaccine coverage. PWDVax is composed of two components: the acellular component, which is a polyvalent fimbria-toxin multiepitope fusion antigen (MEFA) monomer protein, and a cellular component, which carries an F4-fimbrial *E. coli* strain and an F18-fimbrial strain to express GM_1_-binding AB_5_ holotoxin-structured fimbria-toxin MEFA. The fimbria-toxin MEFA monomer consists of a chimeric LT A1 peptide (that has backbone epitopes substituted with functional epitopes of fimbriae F4 and F18 and toxins LT, STa, STb, and Stx2e), the LT A2 peptide, and LT B subunit (one copy B subunit), forming a 1A:1B peptide. The AB_5_ holotoxin-structured fimbria-toxin MEFA contains native LT genes (*eltAB*) signal peptides, the chimeric A1 peptide, the LT A2 peptide, the native LT cistron structure, and an LT B pentamer. Different colors in the diagram and secondary structure models indicate epitopes of different ETEC fimbriae and toxins.

Two chimeric fimbria-toxin MEFA genes coding a monomeric fimbria-toxin MEFA protein and a GM_1_-binding holotoxin-structured fimbria-toxin protein were generated (Fig. 1). The monomeric fimbria-toxin MEFA gene consisted of an ETEC native LT B subunit gene segment (*eltB*, without the signal nucleotides) and a chimeric LT A subunit gene segment (mutant *eltA*, without the signal sequence), and the two segments fused as a single gene (without the cistron gene structure between the B subunit and the mutant A subunit segment). The chimeric LT A segment had eight epitopes substituted with epitopes from F4 adhesin FaeG (two epitopes), F18 adhesin FedF (two epitopes), STa toxoid STa_N11S_ (two copies), STb, and Stx2e A subunit. These epitopes (differentiated in colors in Fig. 1), which were demonstrated previously to induce functional antibodies against the fimbria or toxin (38–42), were selected as the representative antigens of the target ETEC fimbriae and toxins. The monomeric fimbria-toxin MEFA gene was cloned into vector pET28α and expressed by *E. coli* BL21 (DE3), and the resultant recombinant strain was designated as 9719.

The GM_1_-binding AB_5_ holotoxin-structured fimbria-toxin MEFA genes were created using ETEC LT genes (*eltAB*) as the template. We substituted the native A1 segment of *eltAB* mutant genes (with the 192nd arginine replaced with glycine for AB_5_ toxoid LT_R192G_) with the chimeric A1 segment of the monomeric fimbriae-toxin MEFA gene and constructed *eltAB*-like AB_5_ holotoxin-structured fimbria-toxin MEFA genes. The resultant construct retained the *eltAB* cistron structure between the chimeric A subunit gene and the B subunit gene (*eltB*), as well as the native signal sequences of each subunit gene to code an LT-like holotoxin-structured fimbria-toxin MEFA protein. The AB_5_ fimbria-toxin MEFA genes were cloned into vector pBR322 and expressed by avirulent F4-fimbrial *E. coli* isolate 1836-2 or an F18-fimbrial strain 8532, leading to two vaccine strains, 9938 and 9957. *E. coli* DH5α also hosted the AB5 fimbria-toxin MEFA genes for recombinant strain 9928.

**Fig 2:**
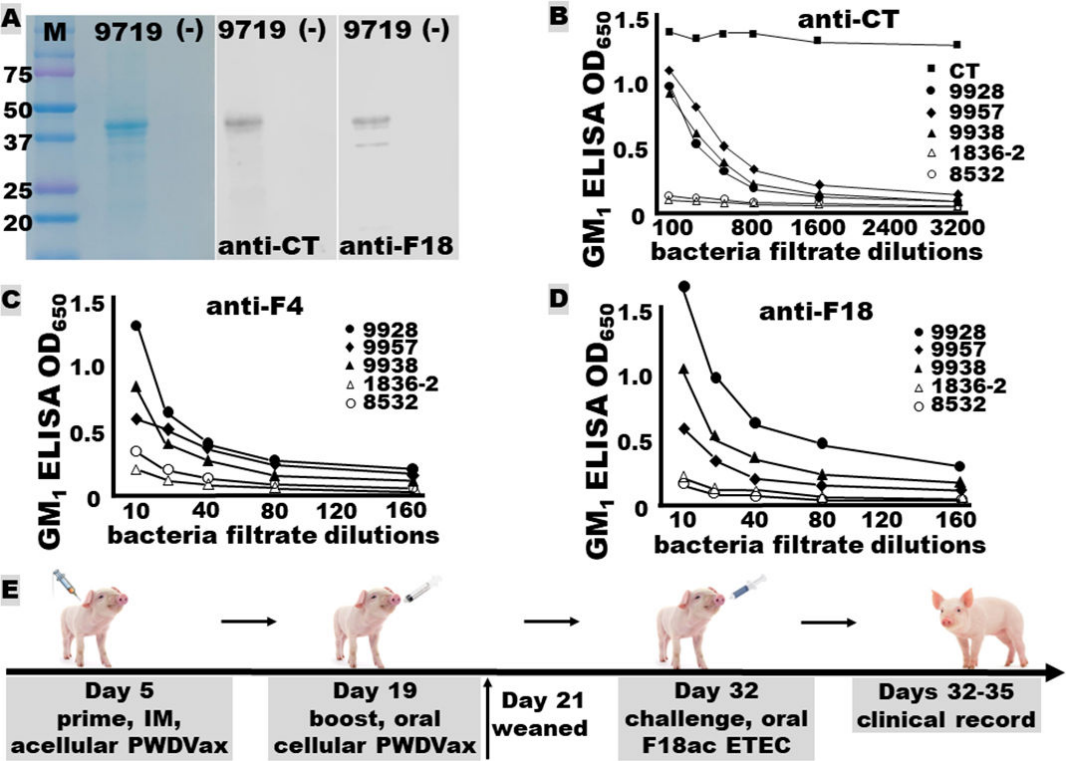
PWDVax vaccine antigen monomeric or holotoxin-structured fimbria-toxoid MEFA expression and detection, and vaccination and challenge schedule. (**A**) SDS-PAGE Coomassie blue staining and Western blot detection (with anti-CT and anti-F18 polyclonal antibodies) of the acellular component antigen, fimbria-toxoid MEFA monomer recombinant protein (9719). (**B**) GM_1_ ELISA with anti-CT polyclonal antibodies to detect the cellular component antigen, holotoxin-structured AB_5_ fimbria-toxoid MEFA expressed and secreted by the recombinant vaccine strain (from bacterial filtrates). (**C**) GM_1_ ELISA with anti-F4 polyclonal antibodies to detect the cellular component antigen. (**D**) GM_1_ ELISA with anti-F18 polyclonal antibodies to detect the cellular component antigen. (**E**) Outline of the heterologous prime-boost immunization schedule and the F18 ETEC challenge study. CT, cholera toxin; 9928, *E. coli* DH5α expressing the holotoxin-structured fimbria-toxoid MEFA; 9957, the F18 fimbrial vaccine strain; 9938, the F4 fimbrial vaccine strain; 1836-2, avirulent F4 fimbrial host strain (host strain of 9938); 8532, avirulent F18 fimbrial host strain (host strain of 9957).

The cloning of the monomeric fimbria-toxin MEFA gene and the AB_5_ fimbria-toxin MEFA genes was confirmed with DNA sequencing. The expression of the fimbria-toxin MEFA monomer protein (~41 kDa) was verified in Western blot with antibodies specific to cholera toxin (CT) (LT homolog) or F18 (Fig. 2A). GM_1_ enzyme-linked immunosorbent assay (ELISA) with polyclonal antibodies specific to CT detected a protein from the bacterial growth filtrates of the two vaccine strains (9938 and 9957) (as well as strain 9928) but not the two vaccine host strains (1836-2 and 8532), suggesting the LT-like holotoxin-structured fimbria-toxin MEFA protein was expressed and secreted by the two vaccine constructs. The expression and secretion of LT-like holotoxin-structured fimbria-toxin MEFA protein from bacterial filtrates of the two vaccine strains (9938 and 9957) was confirmed in GM_1_ ELISAs with anti-CT antibodies (Fig. 2B), as well as F4 (Fig. 2C) or F18 (Fig. 2D) polyclonal antibodies. Bacteria adherence assays showed that vaccine strains 9938 and 9957 bound to porcine intestinal cells IPEC-J2 equally effectively as their respective host strain, 1836-2 or 8532.

F4-fimbrial vaccine strain 9938 and F18-fimbrial vaccine strain 9957 showed nearly identical growth curves and viability (up to five passages were tested). By mixing these two strains at an equal ratio (volume), we prepared the cellular component of a PWD vaccine candidate. A combination of this cellular vaccine component with the acellular component (recombinant fimbria-toxin MEFA monomer protein), a two-component PWD vaccine candidate, PWDVax, was produced. PWDVax targets both F4 and F18 fimbriae and four ETEC toxins (LT, STa, STb, Stx2e) and is expected to protect against ETEC strains expressing either F4 or F18 fimbria and any of the four toxins, thus pathotypes associated with a vast majority of PWD clinical cases (Fig. 1).

### A heterologous prime-boost immunization with two-component vaccine candidate PWDVax elicited antibody responses to fimbriae (F4 and F18) and ETEC toxins (LT, STa, STb, and Stx2e)

PWD vaccine candidate PWDVax is intended to be administered in a heterologous prime-boost immunization schedule (Fig. 2E). This includes a primary intramuscular injection with vaccine acellular component, fimbria-toxin MEFA monomer protein to induce host systemic immunity and an oral boost with vaccine cellular component, live *E. coli* bacteria to colonize pig small intestines and deliver the holotoxin-structured fimbria-toxin MEFA that binds to host GM receptors on pig intestinal epithelial cells to mount host local mucosal immunity.

**Fig 3:**
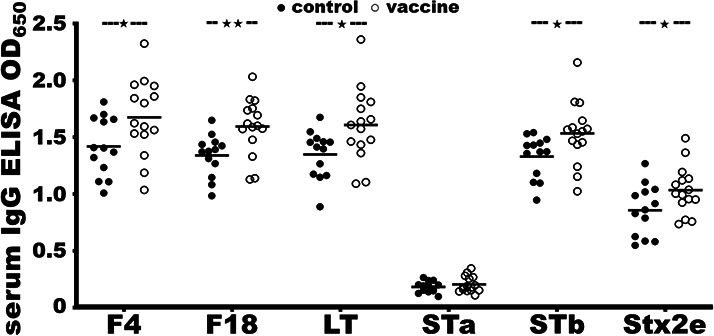
The ELISA OD_650_ values to measure IgG responses to F4, F18, LT, STa, STb, and Stx2e from pig serum dilutions of the control group (●; *n* = 13) or the vaccine group (○; *n* = 15). Serum samples were collected on day 19, 2 weeks after the primary intramuscular immunization with the fimbria-toxin MEFA monomer protein or PBS, adjuvant with dmLT. Recombinant protein of F4 fimbrial subunit FaeG, F18 fimbrial subunit FedF, cholera toxin (CT), MBP-STb, or MBP-Stx2eA, 100 ng per well, or STa-ovalbumin conjugates (10 ng per well) were used as the ELISA coating antigen. Pig serum dilution (1:400), HRP-conjugated goat-anti-pig IgG (1:5,000), and TMB substrate were used to measure OD_650_ values. Bars indicate the mean OD readings in the group. An independent samples *t*-test was performed to calculate *P* values. ★ and ★★ show a *P* value of <0.05 and <0.01, respectively.

Serum samples collected from each piglet 14 days after the primary immunization with the fimbrial-toxin MEFA monomer protein showed a significant rise in IgG responses to F4 and F18 fimbriae and toxins LT, STb, and Stx2e (Fig. 3). The ELISA OD_650_ values specific to F4, F18, LT, STb, and Stx2e from the sera of the immunized piglets were 1.68 ± 0.34, 1.59 ± 0.25, 1.61 ± 0.32, 1.53 ± 0.28, and 1.03 ± 0.21, respectively, significantly greater than the OD values to the antigen from the control sera, 1.42 ± 0.26 (*P* < 0.05), 1.34 ± 0.18 (*P* < 0.01), 1.35 ± 0.21 (*P* < 0.05), 1.33 ± 0.19 (*P* < 0.05), and 0.86 ± 0.23 (*P* < 0.05). Serum IgG response to STa was not detected in the two groups. No IgA responses were detected from pig serum samples.

Antigen-specific IgG responses from the sera collected from each piglet on day 5 (before the prime) showed no differences between the two groups except for Stx2e. The serum IgG ELISA OD values from the immunization group and the control group to F4 (0.97 ± 0.13, 0.88 ± 0.10; *P* = 0.64), F18 (0.96 ± 0.14, 0.91 ± 0.17; *P* = 0.45), LT (0.99 ± 0.21, 0.85 ± 0.20; *P* = 0.12), STa (0.24 ± 0.08, 0.20 ± 0.12; *P* = 0.32), and STb (1.05 ± 0.16, 1.17 ± 0.13; *P* = 0.06) were not significantly different. The OD values to Stx2e from the control piglets were greater than the immunized group (0.82 ± 0.13 vs 0.61 ± 0.14). However, after the prime, serum anti-STx2e IgG was significantly increased (from 0.61 to 1.03) in the immunized group, whereas the anti-STx2e IgG in the control pigs were the same (0.82 and 0.86). The OD values to the target antigens from the sera collected on day 32 (before the F18 ETEC challenge) showed that the immunized piglets were greater than the control piglets, though not statistically significant.

**Fig 4:**
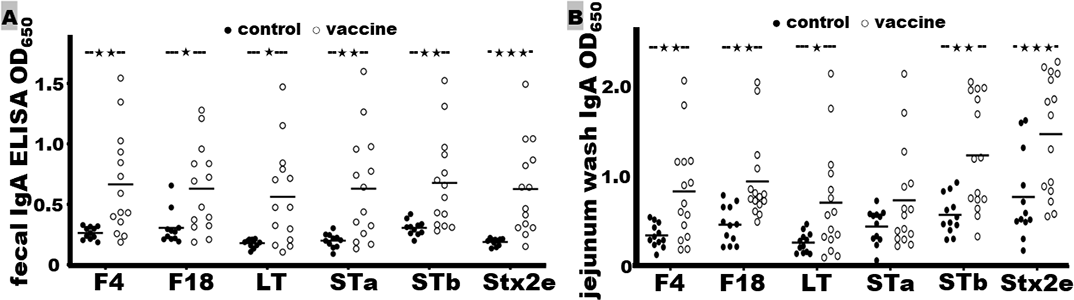
The ELISA OD_650_ readings to measure sIgA responses to F4, F18, LT, STa, STb, and Stx2e from pig fecal suspension samples (**A**) or jejunum washes (**B**) of the control group (●) or the vaccine group (○). (**A**) Fecal samples collected before or after euthanasia at necropsy were suspended in fecal reconstitution buffer (1 g in 3 mL buffer; 1:4 dilution), and supernatants were collected and used in ELISAs to examine sIgA responses to the vaccine antigens. (**B**) Jejunum washes collected at necropsy were used in ELISAs to examine sIgA responses to the vaccine antigens. Bars indicate the mean OD readings in the group. A Welch *t*-test was used to calculate *P* values for fecal IgA of F4, STa, STb, Stx2e, and jejunum IgA of F4; Mann-Whitney *U*-test for fecal IgA of F18, LT, and jejunum IgA of F18, LT, STa, STb, and Stx2e; ★, ★★, and ★★★ indicate a *P* value of <0.05, <0.01, and <0.001, respectively.

Antigen-specific IgA responses were detected from the pigs after the oral booster with the cellular component of PWDVax (Fig. 4). The fecal suspension samples collected at necropsy showed 14 immunized pigs (not sufficient feces were collected from one pig that had water diarrhea) had a significant elevation of secretory IgA (sIgA) antibody responses to the target antigens (Fig. 4A). The ELISA OD_650_ values to F4, F18, LT, STa, STb, and Stx2e sIgA response were 0.67 ± 0.42, 0.63 ± 0.36, 0.56 ± 0.41, 0.63 ± 0.45, 0.68 ± 0.39, and 0.63 ± 0.39, respectively. These OD values were significantly elevated compared to the OD values from the fecal suspension samples of the control piglets (feces were collected from 11 out of 13 control piglets), 0.26 ± 0.05 (*P* < 0.01), 0.31 ± 0.13 (*P* < 0.05), 0.18 ± 0.03 (*P* < 0.05), 0.20 ± 0.06 (*P* < 0.01), 0.31 ± 0.06 (*P* < 0.01), and 0.19 ± 0.04 (*P* < 0.001), respectively.

The jejunum washes collected at necropsy also showed a significant rise in IgA responses after oral boost with the PWDVax cellular component (Fig. 4B). The ELISA OD_650_ readings specific to F4, F18, LT, STb, or Stx2e were 0.84 ± 0.56, 0.95 ± 0.47, 0.71 ± 0.60, 1.24 ± 0.62, and 1.48 ± 0.66, respectively, from the jejunum washes of the immunized piglets. These OD values were significantly higher than the OD readings from the control piglets: 0.35 ± 0.13 (*P* < 0.01), 0.47 ± 0.21 (*P* < 0.01), 0.27 ± 0.11 (*P* < 0.05), 0.58 ± 0.21 (*P* < 0.01), and 0.78 ± 0.49 (*P* < 0.001), respectively. The IgA OD values to STa from the jejunum washes of the immunized piglets were 0.74 ± 0.57, higher but not significantly than the OD from the control piglets (0.45 ± 0.19; *P* = 0.22).

### Heterologous prime-boost immunization with the two-component PWDVax protected weaned pigs from F18 ETEC diarrhea

After oral challenge with F18 ETEC strain 9922 (F18ac, LT, STa, STb, and Stx2e), all 13 control pigs developed diarrhea, 7 with watery diarrhea and 6 with mild diarrhea. In contrast, 10 out of 15 immunized pigs remained healthy; only 1 pig developed watery diarrhea, and 4 showed mild diarrhea (Table 1). PWDVax efficacy against F18 ETEC-associated PWD was calculated at 87.5% against watery diarrhea and 66.7% against any diarrhea.

**TABLE 1:** Clinical outcomes after challenge with an F18 ETEC strain and post-weaning diarrhea (PWD) vaccine candidate PWDVax efficacy against F18 ETEC-associated PWD in pigs

Treatment group	No. of piglets with clinical outcomes/total no. in the group (%)			Efficacy (%) against	
	Normal	Mild diarrhea	Watery diarrhea	Any diarrhea	Watery diarrhea
Control (*n* = 13)	0/13 (0)	6/13 (46.2)	7/13 (53.8)	66.7	87.5
Vaccine (*n* = 15)	10/15 (66.7)	4/15 (26.7)	1/15 (6.7)		

**Fig 5:**
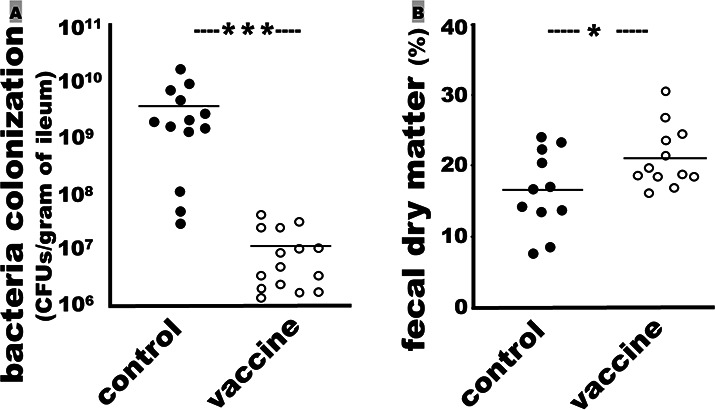
Colonization of the F18 ETEC strain in small intestines and dry matter in feces of the immunized or control pigs after challenge. (**A**) Ileum tissues collected from the control pigs (●; *n* = 13) or the immunized pigs (○; *n* = 15) at necropsy were used to quantify colonization (CFUs per gram) of the F18 ETEC challenge strain (9922; F18ac, LT, STa, STb, and Stx2e) in pig small intestines. (**B**) Dry fecal matter (wt%) from the fecal secretion samples collected before euthanasia at necropsy from the control pigs (●; *n* = 12) or the immunized pigs (○; *n* = 12). Feces were weighed, oven-dried, and weighed again to calculate the dry matter referred to as the initial weight. Bars indicate the mean OD reading in the group. Independent samples *t*-test was performed to calculate *P* values; ★ and ★★★ show a *P* value of <0.05 and <0.001, respectively.

PWDVax conferred protection against ETEC intestinal colonization in weaned pigs. Quantitative colonization studies using pig ileum tissues showed that colonization of the challenge F18 ETEC bacteria in the small intestines was reduced by over 99% in the pigs vaccinated with PWDVax, compared to the control pigs. The challenge bacteria (CFUs per gram ileum tissue) colonized in the small intestines of the immunized pigs were 1.1 ± 1.2 (×10^7^), significantly lower than the CFUs from the control pigs (3.3 ± 4.3, ×10^9^; *P* < 0.001) (Fig. 5A). PCR using specific primers confirmed that the colonized *E. coli* bacteria were the F18 ETEC challenge strain.

The fecal dry matter from the pigs immunized with PWDVax was significantly heavier than that of the control pigs (Fig. 5B). The dry matter of the feces collected from the vaccinated pigs (*n* = 12) was 21 ± 4.3 (%), significantly greater than the control pigs (16.2 ± 5.6; *P* < 0.05; *n* = 12). However, the daily body weight gain over the 3 days post-challenge (from challenge on day 32 to the necropsy on day 35) showed no significant differences for pigs in the two groups.

## DISCUSSION

Data from this study showed that heterologous prime-boost immunization with the two-component vaccine candidate PWDVax protected against F18 ETEC-associated PWD. Piglets primed with intramuscular immunization of the PWDVax acellular component (fimbria-toxin MEFA monomer protein) and boosted with the oral inoculation of PWDVax cellular component (two live strains expressing GM_1_-binding holotoxin-structured fimbria-toxin MEFA protein) developed vaccine antigen-specific IgG and IgA antibodies and were protected from ETEC intestinal colonization but also clinical diarrhea after infection with an F18 ETEC challenge strain. These results suggested that PWDVax could be an effective vaccine to prevent PWD, improve swine health and well-being, and reduce economic losses for global swine producers.

Developing vaccines to protect the newly weaned pigs from PWD has encountered challenges historically. Piglets are often weaned at 21 days or even as early as 17 days. Since passive maternal antibodies no longer protect them, and without active immunity, weaned piglets can develop diarrhea within 24–48 h after entering weaning. That requires piglets to be vaccinated at the age of as early as 3 days to develop active immunity at a sufficient level to be protected against diarrhea by the time of weaning. Oral immunization with a cellular vaccine is preferred for mounting host mucosal immunity, and intestinal mucosal immunity is believed to play a more important role in protecting against enteric diseases, including PWD. However, orally administered whole-cell vaccine products were shown to be not very effective at inducing immune responses in very young children (43–45) or animals, particularly piglets orally immunized with F18 fimbrial *E. coli* whole-cell products (34, 35, 46). To overcome this challenge, in this study, we created a two-component PWD vaccine candidate composed of an acellular component and a cellular component and applied a heterologous prime-boost vaccination strategy, an intramuscular injection with the acellular vaccine component as the primary immunization at the age of 3–5 days and followed by an oral booster with the cellular vaccine component before weaning. Results showed that piglets intramuscularly immunized with fimbria-toxin monomer protein at day 5 developed IgG antibody responses to ETEC fimbriae (F4 and F18) and toxins (LT, STb, and Stx2e; a lack of response to STa after prime only is not unexpected because of the STa poor immunogenicity). After the oral booster with the vaccine cellular component, two live strains expressing GM_1_-binding holotoxin-structured fimbrial-toxin MEFA protein, piglets had a significant elevation of IgA responses to the fimbriae (F4 and F18) and toxins (LT, STa, STb, and Stx2e), detected from their fecal samples and jejunum washes (collected at necropsy on day 35, 3 days after the F18 ETEC challenge).

An application of a two-component vaccine product with a heterologous prime-boost vaccination schedule, a primary intramuscular injection of a protein antigen to mount active systemic immunity in the pigs before weaning, in conjunction with an oral booster with a live cellular product to induce antigen-specific intestinal IgA antibodies in pigs after weaning, potentially enables and extends this vaccine product (PWDVax) for effective protection against PWD. While the role(s) played by systemic antibodies derived from the intramuscular immunization of the PWDVax acellular component in protection against F18 ETEC PWD is not defined in the current study since we did not challenge pigs right after weaning, recent studies demonstrated that intramuscular injection of protein-based vaccine candidates (with adjuvant dmLT, a double mutant of ETEC toxin LT) can protect against intestinal colonization of ETEC and other non-invasive enteric pathogens (47, 48). Though ETEC bacteria do not invade pig (or human) intestinal epithelial cells, ETEC toxins enter host epithelial cells and essentially disrupt intestinal cell homeostasis to cause watery diarrhea. Serum antitoxin IgG antibodies elicited from the intramuscular injection of fimbria-toxin MEFA protein can neutralize toxin enterotoxicity intracellularly and perhaps may also reduce ETEC intestinal colonization to some degree since toxins, particularly for LT which enhances ETEC intestinal colonization (24, 25), potentially leading to protection against PWD clinical diarrhea or reduction of disease severity.

To further differentiate protection between systemic immunity from the intramuscularly immunized fimbria-toxin MEFA monomer protein and intestinal mucosal immunity from the orally administered holotoxin-structured fimbria-toxin MEFA, we will need to carry out comparative studies in the future. By using homologous versus heterologous prime-boost immunization: intramuscular prime and boost with the fimbria-toxin MEFA monomer protein, oral prime and boost with live bacteria expressing GM_1_-binding holotoxin-structured fimbria-toxin MEFA, we can examine protection outcomes and compare them with results from the current study—intramuscular prime with the fimbria-toxin MEFA monomer protein and oral boost with live bacteria expressing GM_1_-binding holotoxin-structured fimbria-toxin MEFA. Knowing that an F18 fimbrial *E. coli* strain or purified F18 fimbriae induces little protective immunity against F18 ETEC-associated diarrhea (34, 35) and colonization resistance from an F18-fimbrial avirulent or vaccine strain is not effective against the current F18 ETEC outbreaks in the United States (26). We believe that the protection against the intestinal colonization of the F18 ETEC challenge strain (isolated from a current outbreak) demonstrated in this study is likely attributed to active immunity from PWDVax, perhaps mainly the FedF antigens (F18 adhesin epitopes).

Other limitations of this study include that we only challenged the immunized pigs 2 weeks after weaning. Since weaned pigs can develop diarrhea at any time during the post-weaning period, future studies to challenge weaned pigs at different times will allow us to better evaluate the efficacy of PWDVax. Also, the current study used an F18ac ETEC strain in the challenge study; future studies with an F4 ETEC challenge strain will help us to evaluate PWDVax broad efficacy against PWD. Knowing that unlike F18, F4 fimbriae or an avirulent F4-fimbrial *E. coli* strain elicits anti-F4 immunity and protects against F4 ETEC diarrhea in pigs. We need to include another control group to be immunized with an avirulent F4 *E. coli* strain or the PWDVax F4 fimbrial host strain to exclusively evaluate the protection from the vaccine antigen (fimbria-toxin MEFAs) against F4 ETEC-associated PWD. We would also like to point out that data from the current study are based on piglets from two litters; therefore, future studies with an increased sampling size can further confirm the candidacy of this vaccine candidate. The current study also did not investigate potential interference from existing antibodies to this vaccine, especially considering that F4 fimbria and LT B subunit are the common antigens of vaccines to immunize pregnant sows for passive protection against ETEC-associated neonatal diarrhea. However, we believe interference from passive antibodies (to an entire fimbria or toxin subunit) with PWDVax should be minimal since only epitopes are included in this vaccine product; moreover, a separate immunization study with PWDVax protein component in piglets with or without maternal antibodies (born to sows immunized with ETEC vaccine Porcilis or control phosphate-buffered saline [PBS]) suggested that interference from passive maternal antibodies is not significant (data not published). Additionally, though the ETEC challenge strain was the only ETEC strain isolated from the pigs with PWD and PCR confirmed the presence of ETEC virulence genes (F18ac, LT, STa, STb, and Ste2e), future studies to characterize the pathogenesis of this strain better will be needed.

Importantly, this study demonstrated that PWDVax induces systemic and intestinal mucosal antibodies against two fimbriae (F4 and F18) and four ETEC toxins (LT, STa, STb, and Stx2e) and protects against F18 ETEC-associated PWD, signifying the potential use of this vaccine candidate in preventing PWD, particularly against the emerging F18 ETEC-associated severe PWD outbreaks in the United States. Interestingly, the use of a heterologous prime-boost immunization strategy, intramuscular immunization with a protein-based vaccine component to elicit neonatal immunity followed by a boost with a cellular vaccine component to mount local mucosal immunity, in addition to the application of an epitope- and structure-based vaccine antigen to precisely target virulence determinants and minimize potential interference from passive material antibodies, may be informative in general for the development of effective vaccines against infections in neonates or the very young animals or humans.

## MATERIALS AND METHODS

### Bacteria strains

The bacteria strains and plasmids used for this study are listed in Table 2. *E. coli* BL21 (DE3) CodonPlus strain (Agilent Technologies, Santa Clara, CA, USA) was used to express the fimbria-toxin MEFA monomer protein as the vaccine acellular component. Avirulent F4-fimbrial field strain 1836-2 (25) and F18-fimbrial isolate 8532 (16) were used as the vaccine host strains to produce a GM_1_-binding AB_5_ holotoxin-structured fimbria-toxin MEFA as the vaccine cellular component. F18 ETEC field strain 9922 (F18ac, LT, STa, STb, and Stx2e), which was isolated from a recent severe PWD outbreak in the United States, was acquired from the Veterinary Diagnostic Laboratory at Iowa State University through Dr. Joseph Connor (Carthage Veterinary Service, Ltd) and used in the challenge study to evaluate PWDVax protection against F18 ETEC-associated PWD.

**TABLE 2:** *Escherichia coli* strains and the plasmids used in this study^*a*^

Strains	Relevant properties	Sources
DH5α	*fhuA2*, *Δ* (argF-lacZ), U169, *phoA*, *glnV44*, *φ* 80, *Δ* (*lacZ*)M15, *gyrA96*, *recA1*, *relA1*, *endA1*, *thi-1*, *hsdR17*	Promega
BL21(DE3) CodonPlus	*huA2*, *Δ(argF-lacZ*), *U169*, *phoA*, *glnV44*, *φ80*, *Δ(lacZ)M15*, *gyrA96*, *recA1*, *relA1*, *endA1*, *thi-1*, *hsdR17*	Agilent Technologies
1836-2	Avirulent *E. coli* isolated from pigs (F4, EAST1, and Paa), F4-fimbrial host strain	(25)
8532	Avirulent *E. coli* isolated from pigs (F18), F18-fimbrial host strain	(16)
9719	“Fimbria-toxin MEFA monomer gene + pET28α” in BL21 (DE3) CodonPlus	This study
9928	“GM_1_-binding holotoxin-structured fimbria-toxin MEFA genes + pBR322” in DH5α	This study
9938	9717 plasmid in 1836-2, F4-fimbrial vaccine strain	This study
9957	9717 plasmid in 8532, F18-fimbrial vaccine strain	This study
9922	F18 ETEC field isolate (F18ac, LT, STa, STb, and Stx2e), ETEC challenge strain	This study
		
Plasmid		
pET28α	Protein expression vector	Novagen
pBR322	Gene clone vector	Promega
pPWD_MEFA_mono	“Fimbria-toxin MEFA monomer gene” in pET28α	This study
p9717, pPWD_MEFA_holo	“GM_1_-binding holotoxin-structured fimbria-toxin MEFA genes” in pBR322	This study

^*a*^ *E. coli* BL21 (DE3) CodonPlus strain was used to express the fimbrial-toxin MEFA monomer protein as the acellular component of PWD vaccine candidate PWDVax. Avirulent F4-fimbrial (1836-2) and F18-fimbrial (8532), two *E. coli* isolates were used as vaccine host strains to express the GM_1_-binding holotoxin-structured fimbria-toxin MEFA as the cellular component of PWDVax. F18-fimbrial ETEC strain 9922 (F18ac, LT, STa, STb, and Stx2e) isolated from a recent severe PWD outbreak was used as the pig challenge strain.

### Vaccine acellular component fimbria-toxin MEFA monomer protein construction, expression, and extraction

To construct a chimeric fimbria-toxin gene for the vaccine acellular component antigen, we utilized the ETEC toxin LT A subunit gene (*eltA*; without signal nucleotides) as a backbone and integrated foreign nucleotide segments of interest into the backbone. This *eltA* gene was PCR amplified from porcine ETEC strain 3030-2 (25) and mutated at the nucleotides coding the 192nd amino acid (from arginine to glycine). By retaining the nucleotides coding the two best neutralizing epitopes of the LT A subunit backbone (41), we replaced the backbone nucleotides coding surface-exposed but less immunodominant epitopes with the nucleotides coding two top functional epitopes of F4 major subunit FaeG (39), two epitopes of F18 adhesin subunit FedF (40), toxin domain of STa toxoid STa_N11S_, STb, and Stx2e epitope (42), and then constructed a chimeric LT A subunit gene as we described previously (38). This chimeric gene was synthesized (Genscript Biotech, Piscataway, NJ, USA) and genetically fused to the LT B subunit gene (*eltB*; without signal nucleotides) to code a single peptide named PWD fimbria-toxin MEFA monomer. The PWD fimbria-toxin MEFA monomer gene was cloned into expression vector pET28a (Novogen, Madison, WI, USA), verified with DNA sequencing, and expressed in *E. coli* BL21 (DE3) CodonPlus strain (Agilent).

Recombinant PWD fimbria-toxin MEFA monomer protein expression and purification were carried out as described previously (49, 50). Briefly, a single colony of the recombinant *E. coli* strain was cultured in 200 mL 2× YT medium supplemented with 30 µg/mL Kanamycin (Sigma, St. Louis, MO, USA) at 37°C until OD_600_ reached 0.5–0.7. After four more hours of incubation, bacteria were harvested by centrifugation and lysed with B-PER (bacteria protein extraction reagent, in PBS, Thermo Fisher Scientific, Waltham, MA, USA), lysozyme (10 mg/mL), and sonication. Lysates were collected after centrifugation and used to extract inclusion body proteins with B-PER following the manufacturer’s protocol. Inclusion body proteins were solubilized with solubilization buffer [500 mM CAPS (pH11.0), 30% N-lauroylsarcosine, 1 mM DTT], then refolded and dialyzed with dialysis buffer (50 mM Tris buffer with 0.1 mM DTT for the first two buffer exchanges and without DTT for the final buffer exchange, pH 8.5). Refolded protein was examined in SDS-PAGE with Coomassie blue staining or characterized in Western blot with anti-CT rabbit polyclonal antibodies (Sigma) or anti-mouse sera specific to F18 FedF subunit protein.

### Vaccine cellular component antigen GM_1_-binding AB_5_ holotoxin-structured fimbria-toxin MEFA construction and vaccine strain preparation

To construct *eltAB* genes-like PWD fimbria-toxin MEFA genes for an AB_5_ GM_1_-binding holotoxin-like vaccine antigen to stimulate intestinal mucosal immunity, we used mutant *eltAB* genes (mutated at the 192nd residue of the A1 domain) as the template and substituted its A1 segment with the chimeric A1 of the PWD fimbria-toxin monomer gene. This resulted in the chimeric genes retaining the cistron segment between the A subunit gene (chimeric *eltA*) and the native B subunit gene (*eltB*), as well as the native signal nucleotides of the *eltA* gene and *eltB* gene to code an LT-like GM_1_-binding holotoxin-structured PWD fimbria-toxin protein (Fig. 1). The chimeric genes were cloned into vector pBR322 and verified with DNA sequencing; the plasmid was initially hosted by *E. coli* DH5α strain (9928).

Avirulent F4-fimbrial *E. coli* isolate 1836-2 and F18-fimbrial *E. coli* isolate 8532 were used to host the plasmid carrying the *eltAB*-like PWD fimbria-toxin MEFA genes for two vaccine strains. Porcine *E. coli* field isolates 1836-2 and 8532 were tested in PCR and confirmed for the presence of the F4 *faeG* gene or F18 *fedF* gene and the absence of LT, STa, STb, and Stx2e toxin genes and examined and confirmed in bacteria adherence assays for attachment to pig intestinal cell line IPEC-J2 as we described previously (39). The two resultant vaccine strains were then examined for bacterial adherence to porcine intestinal cell line IPEC-J2. Expression and outer-membrane secretion of the PWD fimbria-toxin MEFA protein from the two vaccine strains and protein binding to receptor GM_1_ were examined in GM_1_ ELISA as we described previously (25). Briefly, overnight culture filtrates from each vaccine strain, each host strain (1636-2, 8532; as the negative control), and strain 9928 were incubated in ELISA plate wells coated with GM_1_ (Sigma). CT (a homolog of LT; Sigma) was also included as a positive control. Anti-CT rabbit polyclonal antibodies (Sigma; 1:1,000) and horseradish peroxidase (HRP)-conjugated goat anti-rabbit IgG (Sigma; 1:3,000) were used as the primary and secondary antibodies. Wells were washed, and OD_650_ values were recorded after 20 min incubation with TMB (3,3′,5,5′-tetramethylbenzidines) Microwell peroxidase substrate (Thermo Fisher). Additionally, GM_1_ ELISAs with anti-F4 (1:1,000) or anti-F18 (1:1,000) mouse sera (and HRP-conjugated goat anti-mouse IgG; 1:3,000) were also used to examine the holotoxin-structured fimbria-toxoid MEFA from each vaccine strain filtrate.

After the expression and secretion of the GM_1_-binding holotoxin-structured PWD fimbria-toxin MEFA from the F4 fimbrial vaccine strain (9938) and the F18 fimbrial vaccine strain (9957) were verified, these two vaccine strains were examined for bacteria growth curve in LB culture and viability using culture up to five passages (by serially diluting culture samples of each pass, plating on LB plates, and counting colony-forming units, CFUs). To verify the two vaccine strains are compatible for culture, they were mixed at an equal ratio of CFUs and co-cultured. Culture samples were serially diluted and plated; after overnight culture, colonies were PCR examined for bacteria composition. After confirming growth compatibility, we combined these two vaccine strains, at a 1:1 ratio, as the cellular component of PWD vaccine candidate PWDVax.

### Heterologous prime-boost immunization of piglets with PWDVax

Sows and boars from the University Swine Research Center were tested with DNA markers to confirm susceptibility to F18 ETEC infection (51). Three susceptible sows were included for breeding, and two of them were used for this study. Sows tested and confirmed with low levels of existing antibodies to F4 fimbria and toxin LT were routinely immunized with Porcilis as recommended by the manufacturer (Merck Animal Health; Rahway, NJ, USA). Ten days before farrowing, the sows were surface-cleaned and transported to the university BSL-II research facility, and each was housed inside a farrowing crate in a separate room. A total of 30 piglets were born by two sows on the same day, 15 piglets each. On day 1, after being wiped with iodine, half of the piglets were swapped between the two litters by coin flipping. To avoid potential sex bias, males and females were kept close to an equal distribution by returning the pig to the litter and picking again if its sex was not proportional in the group. One litter was randomly designated as the control group and the other as the vaccination group. Two piglets in the control group were crushed by the mother after the primary vaccination, leaving 13 piglets in the control group.

A heterologous prime-boost vaccine schedule for PWDVax consisted of primary intramuscular immunization with the vaccine acellular component, recombinant PWD fimbria-toxin MEFA monomer protein, and an oral booster with PWDVax cellular component, a combination of an F4-fimbrial and an F18-fimbrial strains expressing GM_1_ binding holotoxin-structured PWD fimbria-toxin MEFA. On day 5, each piglet in the vaccination group was injected intramuscularly with 200 µg (in 200 µL PBS) PWD fimbria-toxin MEFA monomer protein, and piglets in the control group each received an intramuscular injection of 200 µL PBS. Adjuvant double mutant LT (dmLT, ATCC BEI Resources; 2 µg in 2 µL PBS) was included in the primary immunization in both groups. For the booster immunization on day 19, each piglet in the control group was inoculated orally with 1 mL PBS, and each piglet in the vaccination group was inoculated orally with 5 × 10^9^ CFUs of PWDVax cellular component (in 1 mL PBS), 2.5 × 10^9^ CFUs of each vaccine strain.

### Antigen-specific antibody response examination

Serum samples were collected from each pig on day 5 (just before the primary immunization), day 19 (2 weeks after the primary, just before the oral booster), and day 35 (at necropsy). Fecal samples and jejunum washes were collected from piglets at necropsy (day 35). Feces materials were collected and suspended in fecal reconstitution buffer (10 mM Tris, 100 mM NaCl, 0.05% Tween-20, and 5 mM sodium azide, pH 7.4) supplemented with 0.5 mM phenylmethylsulfonyl fluoride, 1 g feces in 3 mL buffer (1:4 dilution). After centrifugation, fecal suspension supernatants were collected and stored at −80°C until use. Additionally, jejunum segments were washed with PBS and collected as jejunum wash samples.

Sera collected from each piglet were examined for antibody responses specific to vaccine antigens: F4, F18, LT, STb, STa, and Stx2e in ELISAs as we described (38). Briefly, wells of a 96-well microtiter plate coated with 100 ng recombinant protein FaeG (F4 adhesin subunit), FedF (F18 adhesin subunit), CT (Sigma), recombinant protein maltose binding protein-STb (MBP-STb), or MBP-Stx2eA, or 10 ng STa-ovalbumin conjugates were incubated with pig serum dilution (1:400) at 37°C for 1 h, washed with PBS with 0.05% Tween 20 (PBST), and incubated with secondary antibody HRP-conjugated goat-anti-pig IgG or IgA (1:5,000; Thermo Fisher) at 37°C for 1 h. Wells were then washed with PBST, and incubated with TMB peroxidase substrate (Thermo Fisher). After 20 min at room temperature, OD_650_ were measured and readouts were recorded after subtracting background readings.

Fecal suspension samples and jejunum washes were examined for antigen-specific IgA response. Fecal suspension (1:4 dilution) or jejunum wash samples (in PBS) were added to 96-well microtiter plate wells coated with FaeG, FedF, CT, MBP-STb, MBP-Stx2eA, or STa-ovalbumin conjugates, the same as described above.

### Pig challenge with an F18 ETEC strain and vaccine efficacy assessment against PWD

Piglets in each litter were separated from the sow on day 21, and the weaned piglets from the immunization group and the control group were hosted in two separate rooms. On day 32, all pigs were orally inoculated with F18 ETEC field strain 9922 (F18ac, LT, STa, STb, and Stx2e), 2.5 × 10^9^ CFUs (in 2 mL LB broth) to each pig. Pigs were observed twice daily on the first 24 h post-inoculation and every 2–4 h the following two days. Clinical signs, including vomiting, diarrhea, lethargy, dehydration, and death, were examined and recorded. Watery diarrhea was recorded if pigs had a wet rear end and unformed watery stool. Severe diarrhea was defined as watery diarrhea and signs of dehydration (turgor skin, sunken eye orbit, and prominent backbone), and mild diarrhea as pigs with rear end stained with feces and semi-formed stool. Diarrhea was recorded if a pig developed diarrhea in any of the 3 days post-challenge. Piglets were also weighed just before the challenge (day 32) and at necropsy (day 35).

Pigs were euthanized 3 days after the challenge. At necropsy, the content in the small intestine was examined, and ileum segments were collected from each pig. Feces were collected from fecal secretion before euthanasia, and if unsuccessful, then collected from formed feces in the large intestine after euthanasia and suspended in fecal reconstitution buffer (1 g in 3 mL, 1:4 dilution). Fecal suspension supernatants were collected after centrifugation. For unformed feces, colon contents were collected and centrifuged; resultant pellets were weighed and suspended in the buffer (1:4 dilution). Fecal suspension supernatants were stored at −80°C until use for IgA antibody examination. The remaining feces or colon contents (only collected from 12 piglets in each group) were weighed, dried, and weighed to calculate dry fecal matter (% referred to as initial feces or fecal content weight). To collect jejunum washes, we cut a 10-cm-long jejunum, rinsed with 1× PBS to remove fecal content, then added 10 mL fecal reconstitution buffer, inverted a few times with two ends closed, drained the fluid into tubes, centrifuged, and collected supernatants as intestinal wash samples.

Ileum tissues were collected at necropsy to quantify F18 ETEC bacteria colonization in pig small intestines. A segment of 5 cm ileum was rinsed with sterile PBS to remove fecal materials, weighed, ground in sterile PBS (1 g tissue in 9 mL PBS; a dilution of 1:10), serially diluted, and plated on LB agar plates. CFUs were counted after overnight growth at 37°C. Fifty colonies randomly selected were tested in PCR with primers specific to the native LT genes *eltAB* of the F18 ETEC challenge strain (the chimeric fimbria-toxin MEFA genes of the vaccine strains cannot be amplified because of nucleotide substitution).

Vaccine efficacy against F18 ETEC-associated PWD was assessed based on the number of diarrheal pigs in the control group against the vaccine group, using the formula (percentage of diarrhea in the control pigs − percentage of diarrhea in the vaccine group)/(percentage of diarrhea in the control pigs). Vaccine efficacy against watery diarrhea and any diarrhea (watery diarrhea and mild diarrhea) was assessed separately.

### Statistical analyses

Data of pig IgG and IgA antibody responses (in OD values), bacterial intestinal colonization (CFUs per gram ileum tissue; in log_10_), and fecal dry matter (%) were analyzed with R (version 4.2.1; R Foundation for Statistical Computing, Vienna, Austria) and presented in means and standard deviations. Data normality from the immunized and the control groups was examined with the Shapiro-Wilk test; homogeneity of variance was evaluated with Bartlett’s test for the normally distributed data or Levene’s test if one group deviated from normality. The independent samples *t*-test, Welch’s *t*-test, or Mann-Whitney *U*-test was used to calculate *P* values for the data with normality and homogeneity of variance, normality but homogeneity of variance violated, and normality deviated (non-parametric), respectively. A *P* -value < 0.05 indicated significant differences.

## Data Availability

All data are included in this manuscript. Raw data will be made available upon request after manuscript publication.
